# Supplementary material for: Risk factors for catheter-associated bloodstream infection in hemodialysis patients: A meta-analysis
Source: PLoS One. 2024 Mar 27;19(3):e0299715. doi: 10.1371/journal.pone.0299715 (PMC10971780; doi:10.1371/journal.pone.0299715)
Supplement: S1 File — (DOCX) [file pone.0299715.s001.docx]

Age

| Study | OR | OR_LL | OR_UL | log(OR) | SE | Subgroup |
| --- | --- | --- | --- | --- | --- | --- |
| Cheng et al 2019 | 0.591 | 0.322 | 1.084 | -0.525939262 | 0.309658581 | Age≥60 years old |
| Liu et al 2016 | 0.485 | 0.421 | 0.558 | -0.723606388 | 0.07186891 | Age≥60 years old |
| Wan et al 2014 | 0.485 | 0.22 | 1.067 | -0.723606388 | 0.40280069 | Age≥60 years old |
| Huang et al 2017 | 2.807 | 1.304 | 6.04 | 1.032116297 | 0.39106315 | Age＞60 years old |
| Ma* et al 2021 | 2.105 | SE=0.192 | | 0.744315467 | 0.192 | Age＞60 years old |
| Wang et al 2014 | 1.873 | 1.743 | 3.265 | 0.627541423 | 0.160115305 | Age＞60 years old |
| Xiao et al 2018 | 1.837 | 1.734 | 2.364 | 0.608133806 | 0.079062301 | Age＞60 years old |
| Yuan et al 2022 | 2.768 | 1.284 | 5.967 | 1.608133806 | 0.391904103 | Age＞60 years old |
| Donati et al 2020 | 1.041 | 0.955 | 1.089 | 2.608133806 | 0.033495863 | Age＞75 years old |
| Murea et al 2014 | 0.33 | 0.2 | 0.55 | -1.108662625 | 0.258061457 | Age＞75 years old |

Gender

| Study | OR | OR_LL | OR_UL | log(OR) | SE |
| --- | --- | --- | --- | --- | --- |
| Hadian et al 2020 | 2.17 | 1.11 | 4.21 | 0.774727168 | 0.340077202 |
| Donati et al 2020 | 0.421 | 0.139 | 1.276 | -0.865122445 | 0.565564166 |

Diabetes mellitus

| Study | OR | OR_LL | OR_UL | log(OR) | SE |
| --- | --- | --- | --- | --- | --- |
| An et al 2022 | 3.6 | 1.321 | 9.809 | 1.280933845 | 0.511456966 |
| Cao et al 2019 | 2.272 | 1.025 | 5.034 | 0.820660501 | 0.406000583 |
| Cheng et al 2019 | 1.589 | 0.299 | 1.863 | 0.463104888 | 0.466709132 |
| Ding et al 2021 | 2.763 | 1.757 | 4.345 | 1.016317046 | 0.230973967 |
| Huang et al 2017 | 4.189 | 4.32 | 13.407 | 1.432462042 | 0.28890856 |
| Li et al 2021 | 7.035 | 4.294 | 11.528 | 1.950897691 | 0.251928612 |
| Li* et al 2021 | 3.217 | 1.513 | 6.842 | 1.168449248 | 0.384945319 |
| Liu* et al 2021 | 5.527 | 2.338 | 13.062 | 1.709645173 | 0.438880456 |
| Liu et al 2016 | 0.455 | 0.217 | 0.954 | -0.78745786 | 0.37774651 |
| Liu et al 2021 | 2.711 | 1.174 | 6.258 | 0.997317571 | 0.42689896 |
| Luo et al 2019 | 1.682 | 0.983 | 9.491 | 0.519983562 | 0.578441362 |
| Wan et al 2014 | 0.356 | 0.164 | 0.774 | -1.032824548 | 0.395843226 |
| Wang et al 2019 | 2.646 | 2.005 | 7.487 | 0.973049066 | 0.336103092 |
| Wang et al 2022 | 5.940 | 1.207 | 29.236 | 1.781709133 | 0.813077266 |
| Wang et al 2014 | 2.849 | 1.582 | 8.374 | 1.046968056 | 0.425112704 |
| Xiao et al 2018 | 2.894 | 1.528 | 8.347 | 1.062639628 | 0.433148597 |
| Yuan et al 2022 | 2.282 | 1.157 | 4.491 | 0.825052251 | 0.345980854 |
| Zhang et al 2018 | 5.903 | 1.368 | 25.469 | 1.775460696 | 0.745946992 |
| Zhang et al 2019 | 2.61 | 1.221 | 5.578 | 0.959350221 | 0.38754084 |
| Zhang* et al 2019 | 4.947 | 1.772 | 13.809 | 1.598781332 | 0.523778495 |
| Zhao et al 2021 | 3.735 | 1.268 | 11.003 | 1.317747819 | 0.551205895 |
| Zhao et al 2017 | 2.662 | 1.866 | 3.458 | 0.97907772 | 0.157370736 |
| Martin et al 2020 | 2.2 | 1.02 | 4.75 | 0.78845736 | 0.392434181 |
| Grothe et al 2010 | 1.37 | 1.16 | 5.16 | 0.31481074 | 0.380744024 |
| Lemaire et al 2009 | 2.37 | 1.65 | 3.39 | 0.862889955 | 0.183687406 |

Anemia

| Study | OR | OR_LL | OR_UL | log(OR) | SE |
| --- | --- | --- | --- | --- | --- |
| Liu et al 2021 | 4.299 | 1.469 | 12.58 | 1.458382438 | 0.547838356 |
| Liu et al 2016 | 0.467 | 0.248 | 0.877 | -0.761426021 | 0.322213838 |

Renal disease

| Study | OR | OR_LL | OR_UL | log(OR) | SE |
| --- | --- | --- | --- | --- | --- |
| Li *et al 2021 | 2.294 | 1.171 | 4.493 | 0.830297019 | 0.34302616 |
| Martín-Peña et al 2012 | 25.5 | 5.5 | 117.2 | 3.238678452 | 0.780391272 |
| ÇAYLAN et al 2010 | 2.29 | 1.13 | 4.04 | 0.828551818 | 0.325006903 |
| Zanoni et al 2020 | 3.03 | 1.38 | 6.67 | 1.10856262 | 0.401922541 |

History of catheter-associated infection

| Study | OR | OR_LL | OR_UL | log(OR) | SE |
| --- | --- | --- | --- | --- | --- |
| Liu et al 2021 | 3.674 | 1.541 | 8.760 | 1.301280987 | 0.443307232 |
| Taylor et al 2004 | 6.56 | 1.81 | 20.56 | 1.880990603 | 0.619903213 |
| Herc et al 2017 | 2.39 | 1.59 | 3.59 | 0.871293366 | 0.207759741 |

Hypertension

| Study | OR | OR_LL | OR_UL | log(OR) | SE |
| --- | --- | --- | --- | --- | --- |
| Grothe et al 2010 | RR=1.22 | 0.86 | 3.14 | 0.198850859 | 0.330368798 |
| Lemaire et al 2009 | 1.49 | 1.08 | 2.04 | 0.39877612 | 0.162242032 |

Dialysis duration

| Study | OR | OR_LL | OR_UL | log(OR) | SE |
| --- | --- | --- | --- | --- | --- |
| Luo et al 2019 | 2.412 | 0.892 | 10.273 | 0.880456279 | 0.62342047 |
| Wang et al 2020 | 3.016 | 1.268 | 7.175 | 1.10393145 | 0.442133139 |
| Yuan et al 2022 | 3.725 | 1.300 | 10.671 | 1.315066852 | 0.53703202 |

Catheter site

| Study | OR | OR_LL | OR_UL | log(OR) | SE |
| --- | --- | --- | --- | --- | --- |
| Jiang et al 2016 | 1.87 | 1.151 | 3.032 | 0.625938431 | 0.247089627 |
| Liu et al 2016 | 0.279 | 0.096 | 0.804 | -1.276543497 | 0.542155887 |
| Luo et al 2019 | 1.961 | 0.741 | 12.381 | 0.673454547 | 0.71834635 |
| Lv et al 2021 | 2 | 1.079 | 3.708 | 0.693147181 | 0.314912745 |
| Ma et al 2021 | 39.291 | 6.201 | 248.967 | 3.670995485 | 0.941992293 |
| Ran et al 2018 | 1.152 | 1.142 | 1.163 | 0.141499562 | 0.004648409 |
| Wan et al 2014 | 0.448 | 0.205 | 0.981 | -0.802962047 | 0.399378184 |
| Wang et al 2020 | 4.483 | 1.814 | 11.079 | 1.500292465 | 0.461611498 |
| Wang et al 2022 | 4.481 | 1.049 | 19.131 | 1.499846236 | 0.740681818 |
| Wu et al 2020 | 1.39 | 1.048 | 2.385 | 0.329303747 | 0.209774372 |
| Yuan et al 2022 | 3.258 | 1.201 | 8.834 | 1.18111351 | 0.509044227 |
| Zhang et al 2016 | 38 | 6.02 | 239.7 | 3.63758616 | 0.939872674 |
| Zhao et al 2021 | 4.045 | 1.079 | 15.168 | 1.397481551 | 0.674273791 |
| Zhou et al 2020 | 2.235 | 1.014 | 4.926 | 2.397481551 | 1.674273791 |
| Martin et al 2020 | 4.4 | 1.65 | 11.72 | 3.397481551 | 2.674273791 |
| ÇAYLAN et al 2010 | 2.14 | 1.13 | 4.04 | 0.760805829 | 0.325006903 |
| Grothe et al 2010 | 1.56 | 1.50 | 5.65 | 0.444685821 | 0.338313887 |
| Cheng et al 2018 | 2.23 | 1.01 | 4.95 | 5.397481551 | 4.674273791 |
| Zanoni et al 2020 | 3.81 | 1.79 | 8.11 | 1.337629189 | 0.385429145 |
| Murea等 2014 | 0.5 | 0.33 | 0.79 | -0.693147181 | 0.22268885 |

Catheter duration

| Study | OR | OR_LL | OR_UL | log(OR) | SE | Subgroup |
| --- | --- | --- | --- | --- | --- | --- |
| Jiang et al 2016 | 1.56 | 1.128 | 2.168 | 0.444685821 | 0.166673197 | Catheter time＞7d |
| Wang et al 2014 | 8.472 | 6.771 | 12.745 | 2.136766608 | 0.161349554 | Catheter time＞7d |
| Xiao et al 2018 | 8.427 | 6.717 | 12.754 | 2.131440837 | 0.163572276 | Catheter time＞7d |
| Zhou et al 2020 | 3.21 | 1.142 | 9.023 | 1.166270937 | 0.527294837 | Catheter time≥14d |
| Cheng et al 2018 | 3.2 | 1.45 | 7.06 | 1.16315081 | 0.4037963 | Catheter time≥14d |
| Huang et al 2017 | 6.87 | 4.143 | 11.737 | 1.927164106 | 0.265644409 | Catheter time≥15d |
| Ran et al 2018 | 0.924 | 0.87 | 0.982 | -0.079043207 | 0.030892372 | Catheter time≥15d |
| Shen et al 2020 | 0.352 | 0.171 | 0.728 | -1.044124103 | 0.369550381 | Catheter time≥15d |
| Wang et al 2019 | 2.556 | 1.842 | 21.794 | 0.938443537 | 0.630301726 | Catheter time≥15d |
| Wang et al 2022 | 0.085 | 0.01 | 0.754 | -2.465104022 | 1.102756958 | Catheter time≥15d |
| Wu et al 2020 | 0.145 | 0.081 | 1.007 | -1.931021537 | 0.642929015 | Catheter time≥15d |
| Cheng et al 2019 | 3.53 | 0.375 | 3.925 | 1.261297871 | 0.599029491 | Catheter time≥30d |
| Luo et al 2019 | 1.582 | 0.793 | 8.245 | 0.458689869 | 0.597331381 | Catheter time≥30d |
| Li et al 2021 | 5.627 | 3.495 | 9.061 | 1.72757644 | 0.243021968 | Catheter time＞90d |
| Lemaire et al 2009 | 1.85 | 1.35 | 2.55 | 0.615185639 | 0.162242032 | Catheter time＞90d |
| Li*et al 2021 | 2.307 | 1.135 | 4.69 | 0.835947979 | 0.361938758 | Catheter time＞1year |
| Liu*et al 2021 | 2.624 | 1.206 | 5.709 | 0.964699871 | 0.396616015 | Catheter time＞1year |

Number of catheterizations

| Study | OR | OR_LL | OR_UL | log(OR) | SE | Subgroup |
| --- | --- | --- | --- | --- | --- | --- |
| Ma* et al 2021 | 2.110 | SE=0.198 | | 0.746687947 | 0.198 | Catheter times≥2 times |
| Zhang et al 2019 | 5.299 | 1.756 | 15.985 | 1.667518124 | 0.563421502 | Catheter times≥2 times |
| ÇAYLAN et al 2010 | 2.49 | 1.25 | 4.93 | 0.91228271 | 0.350049856 | Catheter times≥2 times |
| An et al 2022 | 3.233 | 1.041 | 10.036 | 1.173410499 | 0.578060418 | Catheter times≥3 times |
| Wang et al 2014 | 9.654 | 5.365 | 15.645 | 2.267372337 | 0.273024235 | Catheter times≥3 times |
| Xiao et al 2018 | 9.645 | 5.356 | 15.654 | 2.266439646 | 0.273599247 | Catheter times≥3 times |

Catheter type

| Study | OR | OR_LL | OR_UL | log(OR) | SE |
| --- | --- | --- | --- | --- | --- |
| Cheng et al 2019 | 1.309 | 0.122 | 1.736 | 0.269263487 | 0.677377003 |
| Wang et al 2020 | 4.039 | 1.636 | 9.972 | 1.395997137 | 0.461103808 |
| Wang et al 2022 | 7.065 | 1.467 | 34.039 | 1.955153016 | 0.80211414 |
| Zhao et al 2021 | 5.765 | 1.77 | 18.773 | 1.751805154 | 0.602408194 |

External hospital tube

| Study | OR | OR_LL | OR_UL | log(OR) | SE |
| --- | --- | --- | --- | --- | --- |
| Ran et al 2018 | 0.908 | 0.888 | 0.928 | -0.0965109 | 0.011239793 |
| Zhou et al 2020 | 1.445 | 0.952 | 2.193 | 0.368109322 | 0.212872631 |

CD4+ cell

| Study | OR | OR_LL | OR_UL | log(OR) | SE |
| --- | --- | --- | --- | --- | --- |
| Ran et al 2018 | 0.903 | 0.829 | 0.985 | -0.102032726 | 0.043985073 |
| Zhou et al 2020 | 0.335 | 0.985 | 0.114 | -1.093624747 | -0.550113059 |
| Cheng et al 2018 | 0.33 | 0.15 | 0.73 | -1.108662625 | 0.403675827 |

ALB

| Study | OR | OR_LL | OR_UL | log(OR) | SE | Subgroup |
| --- | --- | --- | --- | --- | --- | --- |
| Cheng et al 2019 | 1.209 | 0.052 | 1.836 | 0.189793572 | 0.909209401 | ALB＜30g/L |
| Liu et al 2016 | 3.629 | 1.201 | 10.962 | 1.288957128 | 0.564102093 | ALB＜30g/L |
| Luo et al 2019 | 1.832 | 0.968 | 12.354 | 0.605408266 | 0.649618135 | ALB＜30g/L |
| Wan et al 2014 | 2.171 | 1.022 | 4.609 | 0.775187891 | 0.384247302 | ALB＜30g/L |
| Wang et al 2022 | 0.139 | 0.024 | 0.799 | -1.973281346 | 0.894210999 | ALB＜30g/L |
| Yuan et al 2022 | 2.008 | 1.173 | 3.435 | 0.697139202 | 0.274094989 | ALB＜30g/L |
| Zhao et al 2021 | 4.988 | 1.696 | 14.666 | 1.607035028 | 0.550321263 | ALB＜30g/L |
| Huang et al 2017 | 0.163 | 0.087 | 0.306 | -1.814005078 | 0.320835965 | ALB＜35g/L |
| Li et al 2021 | 5.049 | 2.716 | 9.386 | 1.619190204 | 0.316341584 | ALB＜35g/L |
| Lv et al 2021 | 1.721 | 1.154 | 2.567 | 0.542905517 | 0.203955034 | ALB＜35g/L |
| Zhao et al 2017 | 2.006 | 1.387 | 2.625 | 0.69614269 | 0.162739223 | ALB＜35g/L |
| Zhou et al 2020 | 1.505 | 0.591 | 3.833 | 0.408792898 | 0.476935471 | ALB＜35g/L |
| Wang et al 2014 | 10.636 | 7.465 | 20.616 | 2.364244473 | 0.259143378 | ALB＜40g/L |
| Xiao et al 2018 | 10.662 | 7.458 | 20.511 | 2.366686018 | 0.258080114 | ALB＜40g/L |

1. reactive protein

| Study | OR | OR_LL | OR_UL | log(OR) | SE |
| --- | --- | --- | --- | --- | --- |
| Cao et al 2019 | 1.725 | 1.45 | 2.051 | 0.54522705 | 0.088460184 |
| Ding et al 2021 | 1.734 | 1.064 | 2.825 | 0.550430878 | 0.249100248 |

Hb

| Study | OR | OR_LL | OR_UL | log(OR) | SE | Subgroup |
| --- | --- | --- | --- | --- | --- | --- |
| Huang et al 2017 | 0.169 | 0.93 | 0.307 | -1.777856564 | -0.282738989 | Hb＜90g/L |
| Zhao et al 2021 | 4.223 | 1.445 | 12.341 | 1.440545776 | 0.54714738 | Hb＜90g/L |
| Zhao et al 2017 | 1.707 | 1.399 | 2.015 | 0.534737444 | 0.093076913 | Hb＜90g/L |
| Wang et al 2014 | 2.634 | 1.063 | 4.268 | 0.968503603 | 0.354604652 | Hb＜100g/L |
| Xiao et al 2018 | 2.643 | 1.062 | 4.267 | 0.971914636 | 0.35478497 | Hb＜100g/L |

Cholesterol

| Study | OR | OR_LL | OR_UL | log(OR) | SE |
| --- | --- | --- | --- | --- | --- |
| Ma et al 2021 | 9.994 | 3.029 | 32.971 | 2.301984913 | 0.609029555 |
| Zhang et al 2016 | 0.1 | 0.03 | 0.32 | -2.302585093 | 0.603858065 |

PCT

| Study | OR | OR_LL | OR_UL | log(OR) | SE |
| --- | --- | --- | --- | --- | --- |
| Wang et al 2020 | 1.044 | 1.028 | 1.061 | 0.043059489 | 0.008060381 |
| Zheng et al 2018 | 1.08 | 1.32 | 1.563 | 0.076961041 | 0.043105948 |

Inadequate hand hygiene

| Study | OR | OR_LL | OR_UL | log(OR) | SE |
| --- | --- | --- | --- | --- | --- |
| Wang et al 2022 | 17.289 | 2.039 | 146.573 | 2.850070461 | 1.090577578 |
| ÇAYLAN et al 2010 | 3.02 | 1.53 | 5.95 | 1.105256831 | 0.346460072 |

APACHE II scores

| Study | OR | OR_LL | OR_UL | log(OR) | SE |
| --- | --- | --- | --- | --- | --- |
| Ran et al 2018 | 0.882 | 0.863 | 0.901 | -0.125563223 | 0.010992491 |
| Zhou et al 2020 | 2.43 | 1.042 | 5.667 | 0.887891257 | 0.432019881 |
| Cheng et al 2018 | 2.4 | 1.04 | 5.51 | 0.875468737 | 0.425342834 |

Poor patient hygiene

| Study | OR | OR_LL | OR_UL | log(OR) | SE |
| --- | --- | --- | --- | --- | --- |
| Taylor et al 2004 | 3.48 | 1.74 | 7.33 | 1.247032294 | 0.366859797 |
| Samani et al 2014 | 1.399 | 1.184 | 2.008 | 0.335757696 | 0.134755272 |
